# Supplementary material for: Microwave-assisted synthesis of metal-organic chalcogenolate assemblies as electrocatalysts for syngas production
Source: Commun Chem. 2023 Mar 1;6:43. doi: 10.1038/s42004-023-00843-3 (PMC9977941; doi:10.1038/s42004-023-00843-3)
Supplement: Supplementary file 2 — Supporting information [file 42004_2023_843_MOESM2_ESM.pdf]

Supporting Information

Microwave-assisted synthesis of metal-organic chalcogenolate assemblies as electrocatalysts for syngas production

AUTHORS: Hannah Rabl, Stephen N. Myakala, Jakob Rath, Bernhard Fickl, Jasmin S. Schubert, Dogukan H. Apaydin*, Dominik Eder

Institute of Materials Chemistry TU Wien, Getreidemarkt 9, 1060 Vienna

Supplementary Figure 1. below shows the ATR-FTIR spectra for [AgSePh]$_\infty$ and [AgSPh]$_\infty$.

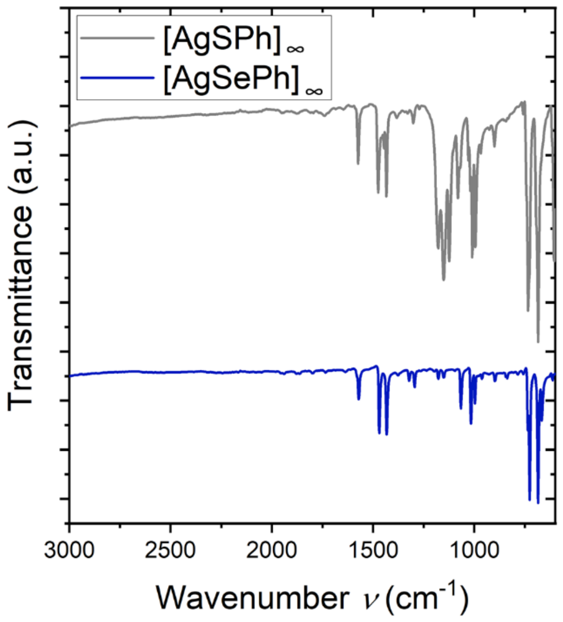

Supplementary Figure 1. ATR-FTIR spectra of the microwave synthesized MOCHAs [AgSePh]$_\infty$ and [AgSPh]$_\infty$. ATR-FTIR spectra of [AgSPh]$_\infty$ (grey) and [AgSePh]$_\infty$ (blue).

The results related to elemental ratio studies are shown in **Supplementary Table 1.**

Supplementary Table 1. Theoretical and experimental elemental ratios. Experimental values were obtained by EDX and XPS measurements.

MOCHA	Carbon (at%)			Metal (at%)			Chalcogenide (at%)		
	Theory	EDX	XPS	Theory	EDX	XPS	Theory	EDX	XPS
[AgSePh]$_\infty$		92.4	78.6		2.5	7.1		5.0	5.1
[AgSPh]$_\infty$	80.0	91.2	70.5	6.7	4.6	10.1	13.3	4.2	9.7

Theoretical calculations reveal 80 at% carbon in a typical MOCHA structure, EDX (Supplementary Figure 2a and 2b) measurements overestimated this value by roughly 10 % for both MOCHA types [AgSePh]$_\infty$ and [AgSPh]$_\infty$. XPS measurements (Supplementary Figure 3a and 3b) of [AgSPh]$_\infty$ however, revealed an accurate estimation of carbon ratio. While EDX

measurements underestimated the metal and chalcogenide ratios for $[AgSePh]_\infty$ and $[AgSPh]_\infty$, these values both reached 10% in case of $[AgSPh]_\infty$.

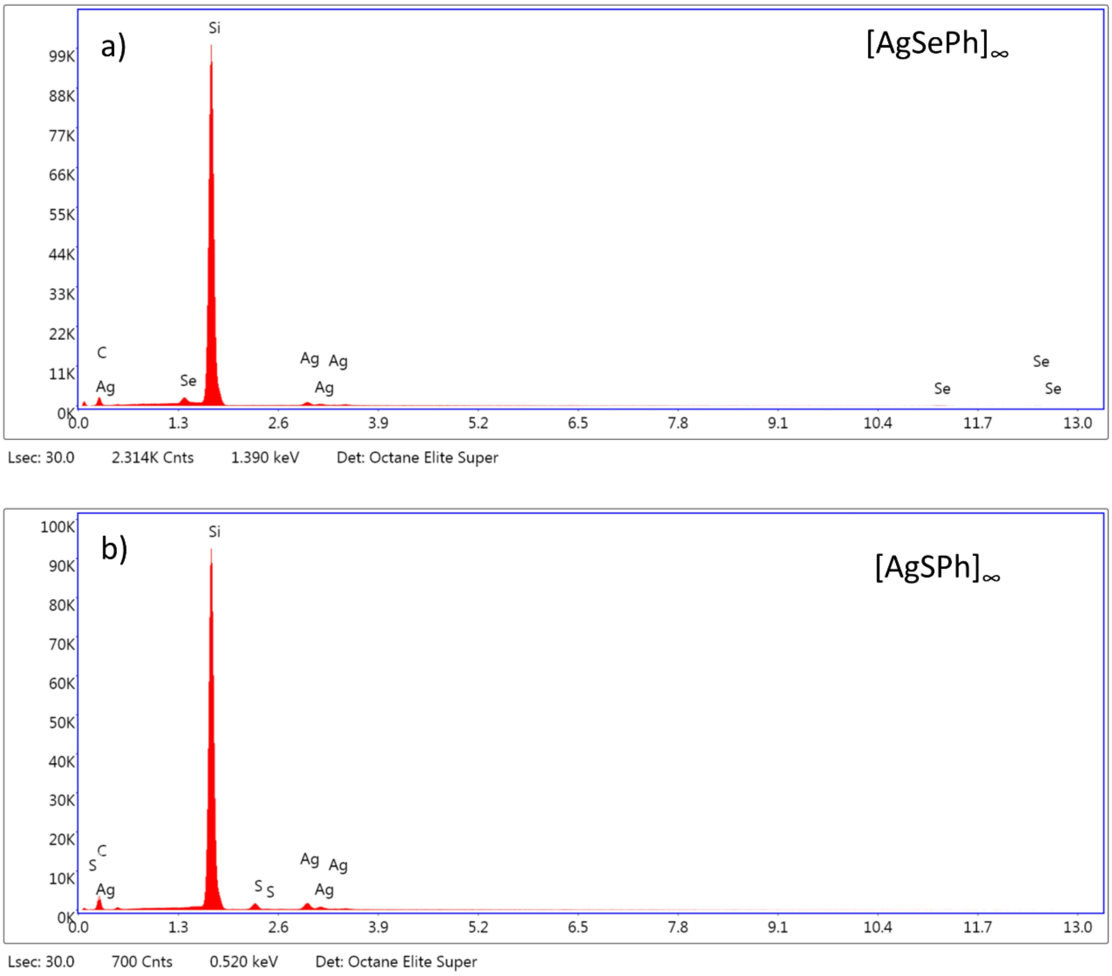

Supplementary Figure 2. EDX spectrum of a) $[AgSePh]_\infty$ and b) $[AgSPh]_\infty$.

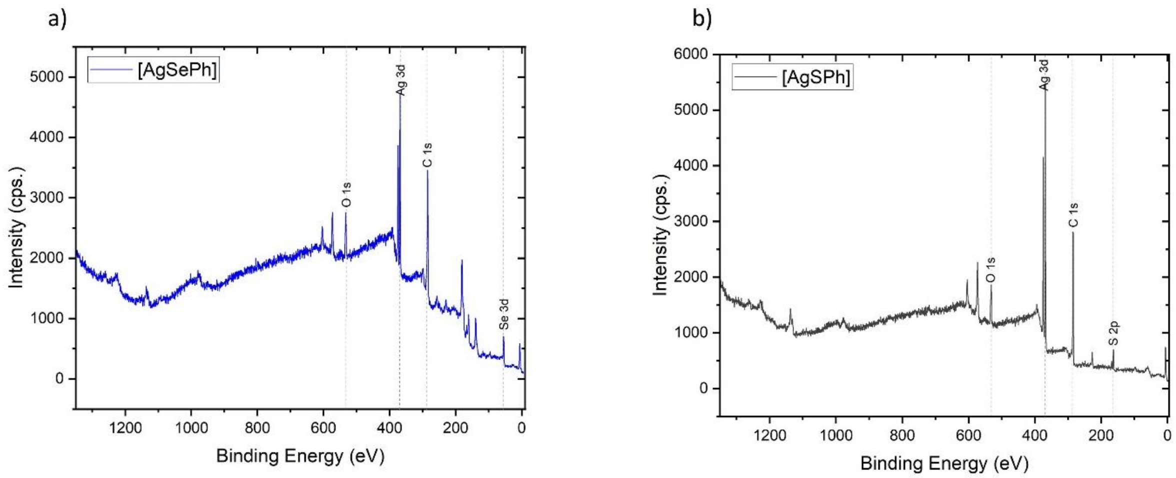

Supplementary Figure 3. XPS spectrum of a) $[AgSePh]_\infty$ and b) $[AgSPh]_\infty$.

In the following three **Supplementary Figures 4-6**, the results obtained during stability testing, performed on $[AgSePh]_\infty$ upon elongated time intervals are summarized.

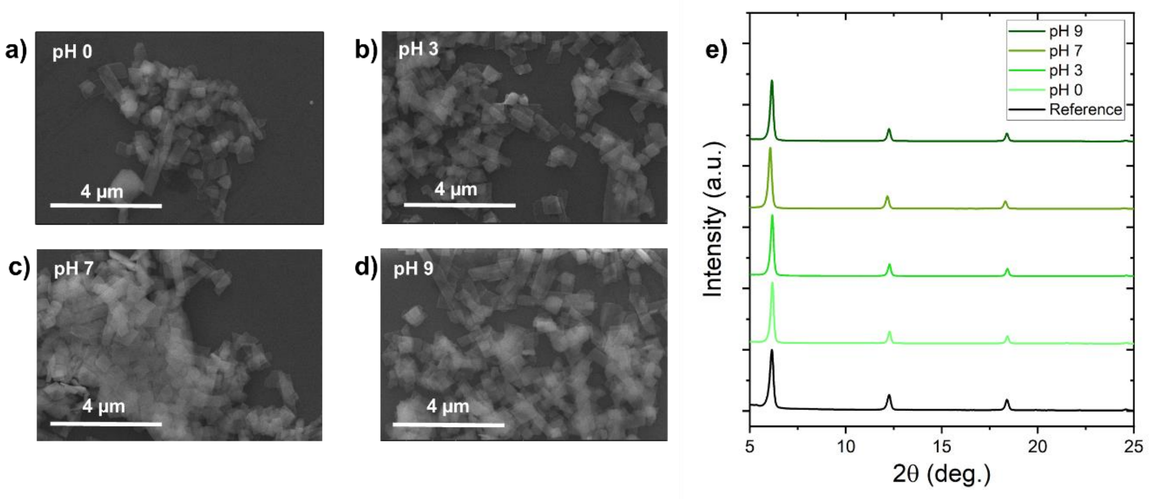

Supplementary Figure 4. Results of stability testing in various pH media. SEM images of MOCHA after being stored at room temperature in **a)** pH 0, **b)** pH 3, **c)** pH 7 and **d)** pH 9 for one week. **e)** XRD pattern of MOCHA after pH stability testing.

The SEM images of MOCHA after exposure to various media with different pH, reveal a wide stability of MOCHA. Also, the XRD pattern display the characteristic peaks, corresponding to the {002}, {004} and {006} crystallographic plane.

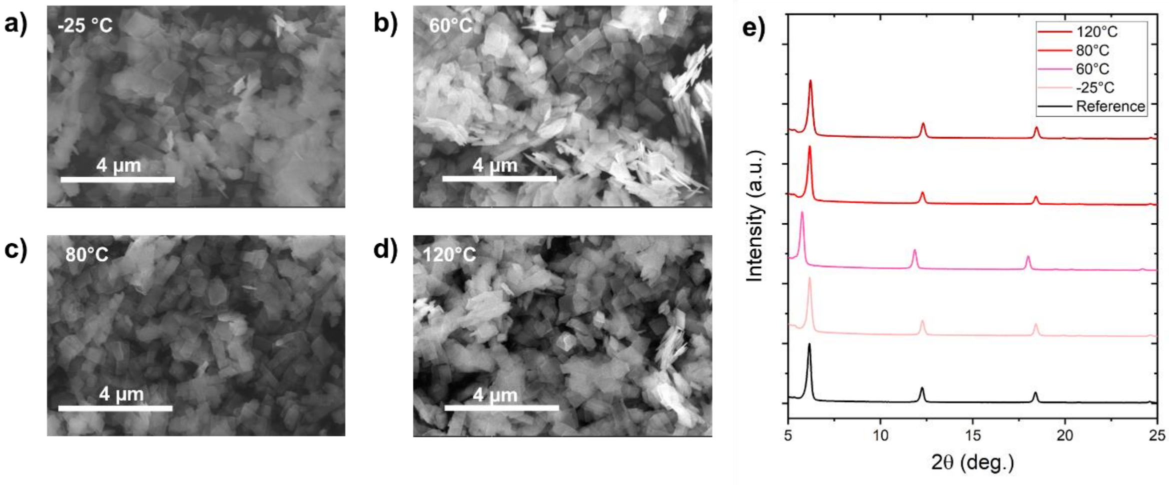

Supplementary Figure 5. **Results of stability testing in various temperatures**. SEM images of MOCHA after being stored at **a)**-25°C, **b)** 60°C, **c)** 80°C and **d)** 120°C for one week. **e)** XRD pattern of MOCHA after temperature stability testing.

As also upon pH, we report a wide stability of MOCHA upon temperatures from -25 °C to 120°C. In fact Popple *et al.* reported stability of $[AgSePh]_\infty$ until 200°C[1]. Hereby benzeneselenol is reported to dissociate at temperatures beyond 200°C[1]. This process is known to be reversible, as shown by the tarnishing method[2].

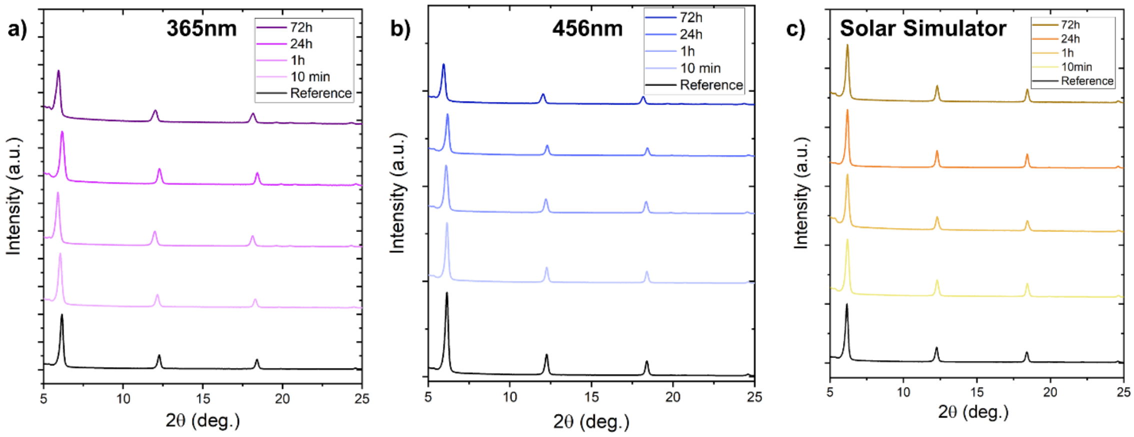

Supplementary Figure 6. XRD pattern of MOCHA after stability testing upon light illumination. Chosen light sources were a **a)** 365 nm lamp, **b)** 456 nm lamp and **c)** solar simulator.

MOCHA proved to be stable upon irradiation with various light sources. XRD measurements reveal the characteristic pattern. We noticed a change in appearance, as the yellow $[AgSePh]_\infty$ powder darkened upon irradiation with all light sources. This effect was described also by Ivanov *et al.* who were working on polyconjugated structures such as polyphene sulphide[3]. As no significant change in mechanical and structural properties was noted in their study[3], we believe that a similar observation is made with MOCHAs here. Light irradiation might cause partial-oxidation to the MOCHA material, without further destroying and influencing structural, mechanical or electronic properties.

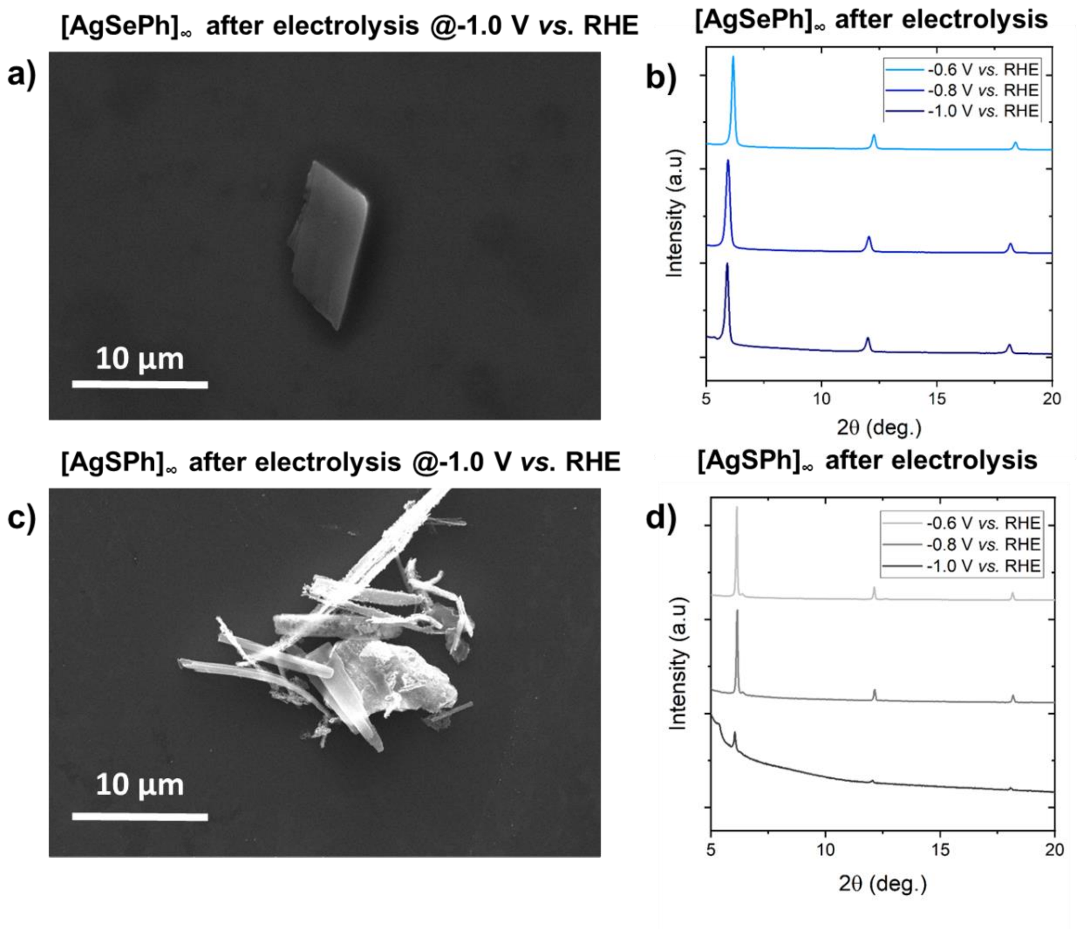

Supplementary Figure 7. Stability test on [AgSePh]$_\infty$ and [AgSPh]$_\infty$ upon electrolysis. a) SEM image of [AgSePh]$_\infty$ sonicated off the CP substrate after electrolysis. **b)** XRD pattern of [AgSePh]$_\infty$ after electrolyses. **c)** SEM image of [AgSPh]$_\infty$, sonicated off the CP substrate after electrolysis. **d)** XRD pattern of [AgSPh]$_\infty$ after electrolyses.

After performed electrolysis, parts of the MOCHA / CP electrode which were immersed in the electrolyte were cut and suspended in isopropanol. After removal of the solvent SEM and XRD measurements revealed remaining MOCHAs after all tested electrolysis conditions. As SEM images in **Supplementary Figure *7*. a** and **c** show, was [AgSePh]$_\infty$ as also [AgSPh]$_\infty$ present after electrolysis at -1.0 V *vs*. RHE. XRD pattern of [AgSePh]$_\infty$ show under all tested overpotentials the characteristic peaks. Measurements on [AgSePh]$_\infty$ at low overpotentials (-0.8 V and -0.6 V *vs*. RHE) also clearly show remains of MOCHA. After electrolysis at -1.0 V *vs*. RHE however only weak diffractions of [AgSPh]$_\infty$ could be observed. This however, might be an effect of the huge background, visible in that measurement. We believe [AgSPh]$_\infty$ to be stable also upon harsher potentials, as SEM images display [AgSPh]$_\infty$ and also weak diffractions can be observed in the XRD pattern.

References

1. Popple, D. C., Schriber, E. A., Yeung, M. & Hohman, J. N. Competing Roles of Crystallization and Degradation of a Metal-Organic Chalcogenolate Assembly under Biphasic Solvothermal Conditions. *Langmuir* **34**, 14265–14273 (2018).
2. Trang, B. *et al.* Tarnishing Silver Metal into Mithrene. *J. Am. Chem. Soc.* **140**, 13892–13903 (2018).
3. Ivanov, V. B., Bitt, V. V., Solina, E. V. & Samoryadov, A. V. Reversible and Irreversible Color Change during Photo and Thermal Degradation of PolyphenyleneSulfide Composite. *Polymers* **11**, 1579 (2019).
